# Outcomes of Isolated Biceps Tenodesis/Tenotomy or Partial Rotator Cuff Repair Associated with Biceps Tenodesis/Tenotomy for Massive Irreparable Tears: A Systematic Review

**DOI:** 10.3390/jcm12072565

**Published:** 2023-03-29

**Authors:** Jules Descamps, Elliott Kierszbaum, Marie Protais, Blandine Marion, Pierre-Alban Bouché, Florence Aïm

**Affiliations:** 1 Hôpital Lariboisière, APHP, Paris 75010, France; 2 Clinique Blomet Paris Ramsay, 75015 Paris, France; 3 Hôpital Saint Antoine, 75012 Paris, France; 4 Bone-and-Joint Infections Referral Center, Groupe Hospitalier Diaconnesses Croix Saint-Simon, 75020 Paris, France

**Keywords:** irreparable massive rotator cuff tear, partial cuff repair, isolated biceps tenotomy/tenodesis

## Abstract

Irreparable large to massive rotator cuff tears (MIRCTs) are a prevalent cause of shoulder pain and dysfunction, and nonoperative treatment may not always be effective. Various surgical options exist, with isolated biceps tenotomy/tenodesis (BT) or arthroscopic partial repair with associated biceps tenotomy/tenodesis (PCR-BT) being the most common. The aim of this study was to systematically review the available data on the clinical and functional outcomes of BT and PCR-BT in patients with MIRCTs. Methods: MEDLINE, Embase, and CENTRAL databases were searched for studies on the treatment of MIRCT. We included studies with BT or PCR-BT with a minimum follow-up of 24 months. The MINORS (Methodological Index for Nonrandomized Studies) score was used to assess study quality. Outcomes included were the visual analog scale for pain, functional scores such as Constant-Murley and American Shoulder and Elbow Surgeons, range of motion, radiological measurements, and complications. Results: A total of 1101 patients (506 had a BT and 595 had a PCR-BT) from 22 studies were included (cases series = 13, case–control = 7, randomized control trial = 1, prospective cohort study = 1). The mean MINORS score was 13.2 ± 3.2. The mean age and follow-up were 67 ± 6.8 years and 4.58 ± 1.1 years (range, 2, 12), respectively. The VAS improvement showed at the last follow-up for PCR-BT (range, 1.97, 5.8) and BT (range, 4, 6.1). CMS was improved at the final follow-up for PCR-BT (range, 13, 47.6) and BT (range, 10.8, 28). Regarding the ASES, it has demonstrated significant improvements for PCR-BT (range, 31.81, 44.8) and BT (range, 30,45.8). For forward flexion, PCR-BT showed improvement (range, −14°, 59.4°), as well as the BT group (range, 2°, 27.9°). Conclusions: This systematic review demonstrated that both BT and PCR-BT improve functional outcomes and reduce pain at midterm follow-up for MIRCT. Since we know that a failed cuff repair would worsen the shoulder, it might be beneficial in terms of the risk–benefit ratio to not repair in certain patients with MIRCT.

## 1. Introduction

Rotator cuff tears (RCTs) are a common cause of shoulder dysfunction and pain. RCTs are considered irreparable when the tears cannot be completely repaired to their insertion using conventional techniques [1]. Other authors consider tears with a chronic acromiohumeral distance (AHD) of less than 7 mm to be irreparable [2]. RCTs are called massive RCTs (MRCTs) historically when the tear is ≥5 cm in diameter [3] or there is a complete tear of 2 or more tendons [4]. 

Conservative treatment, such as physical therapy, non-steroidal anti-inflammatory drugs, and intra-articular corticosteroid injection, should be the first step in management. It is essential to consider that nonoperative treatment has been successful in many patients with chronic, massive, irreparable rotator cuff tears, but not always [5]. Reverse total shoulder arthroplasty (RTSA) has been proposed as a treatment for pseudoparalytic shoulders even in the absence of osteoarthritis, but may not be recommended for all young patients [6]. In these cases, non-arthroplasty procedures can be performed, such as arthroscopic debridement, a BT, partial repair, interposition of a graft for superior capsular reconstruction [7], balloon spacer arthroplasty [8], lower trapezius transfer, and latissimus dorsi transfer.

Patients with low to moderate strength demand and failed nonsurgical treatment are more likely to be candidates for debridement and BT or partial cuff repair, according to Burnier, Elhassan, and Sanchez-Sotelo [9], who introduced the concept of functionally irreparable rotator cuff tear. Biceps and labrum may contribute to pain in MRCTs [10]. Improvements in pain and function may occur after a simple transection of the tendon of the long head of the biceps with or without tenodesis and cuff debridement. This procedure provides low risks of complications and fast recovery, and does not risk imbalance in a deficient shoulder [11].

Partial repair of MRCTs was first described by Burkhart et al. in 1994 [12]. They developed a margin convergence repair of the anterior and posterior margins of the rotator cuff, leaving the central portion of the tear with the greatest retraction unrepaired. Stable horizontal balance with partial cuff repair results in a stable pivot point for the glenohumeral joint. This restores coronal balance, resulting in a functional deltoid muscle with effective forward elevation. The goal is only to restore a stable fulcrum point for the glenohumeral joint. Several biomechanical and clinical studies have demonstrated effectiveness in reducing tension and strain on the torn tendon and have found satisfactory results of arthroscopic rotator cuff repair using the edge convergence technique [13]. However, a high retear rate of 50% after arthroscopic partial repair has also been reported [14].

A number of clinical studies have investigated the clinical and functional outcomes of arthroscopic debridement and BT or PCR-BT for MIRCTs. To our knowledge, only 2 small studies with direct comparison and a relatively small number of patients (55) have been performed [15,16]. The objective was to systematically review the available data with regard to clinical and functional outcomes of isolated biceps tenotomy/tenodesis (BT) or arthroscopic partial repair with associated biceps tenotomy/tenodesis (PCR-BT) in irreparable large to massive rotator cuff tears (MIRCT). Based on the available evidence, the hypothesis was that functional results and pain relief were superior with the PCR-BT technique.

## 2. Materials and Methods

This systematic review and meta-analysis was conducted in accordance with the Preferred Reporting Items for Systematic Reviews and Meta-Analyses (PRISMA) statement [17] and was registered in PROSPERO (Number CRD42021233800).

### 2.1. Search Strategy

**Figure 1:**
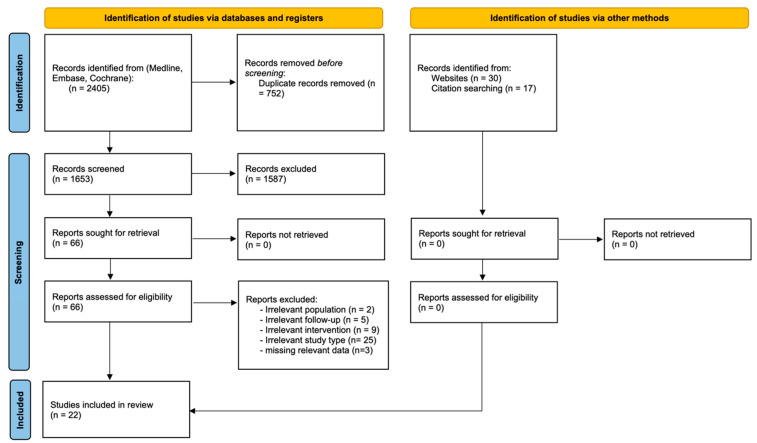
PRISMA 2020 flow diagram for new systematic reviews which included searches of databases and other sources.

The literature search was conducted in three databases: MEDLINE via PubMed, the Cochrane Central Register of Controlled Trials (CENTRAL), and EMBASE. Search terms concerned cuff tear characterization (“irreparable” and “massive”, “cuff”, “tear”) and management (“tenotomy”, “tenodesis”, “biceps”, “partial repair”). A search algorithm was developed for each database: “rotator cuff tear” AND (“tenotomy” OR “tenodesis” OR “biceps” OR “debridement” OR “partial repair”). A time limit was set: no study before 2005 was included. The reference list of each article or report identified by the search and any previously published meta-analyses on the topic of interest were examined. Finally, ongoing trials were identified by searching ClinicalTrials.gov and systematically screening reference lists of systematic reviews and meta-analyses for any additional references (Figure 1). The search began in January 2005 and ended on 31 March 2022.

### 2.2. Selection of Studies

Inclusion criteria were (1) trial evaluated management of (2) irreparable and massive cuff tear; with (3) isolated biceps tenotomy/tenodesis (BT) or partial cuff repair associated with BT (PCR-BT); (4) minimal follow-up of 24 months; (5) in any language; whether (6) the report was published, unpublished, or in press. No (1) systematic reviews, (2) case reports, or (3) reports of expert opinion were included. Relevant trials were selected by two authors (JD and FA) working independently and resolving disagreements by consensus. Excluded studies and reasons for exclusion were listed.

### 2.3. Study Quality Assessment

#### Study

The quality of included studies was evaluated using the MINORS (Methodological Index for Non-Randomized Studies) score [18]. For noncomparative studies, quality was assessed as follows: 0–4, very low quality; 5–8, low quality; 9–12, moderate quality; and 13–16, high quality; and for comparative studies, 0–6, very low quality; 7–12, low quality; 13–18, moderate quality; and 19-24, high quality.

Two authors performed this evaluation, which included discussion to reach a consensus in case of disagreement.

### 2.4. Extraction and Review of the Data

Data in the included studies were extracted to standardized forms by two evaluators (JD and FA), who worked independently from each other. The following were collected: publication date; journal; country; patient demographics (age, gender, type of rupture, type of reparation, follow-up); functional evaluation: (1) Visual Analogue Scale (VAS), (2) Constant-Murley score (CMS), (3) American Shoulder and Elbow Surgeons (ASES), (4) range of motion and radiological data (acromiohumeral distance (AHD)); Hamada classification; and fatty infiltration according to Goutallier classification. The minimum clinically important difference (MCID) was two points for VAS, 10 points for CMS, and 15 points for ASES [19,20,21].

### 2.5. Statistical Analyses

The data were analyzed using RevMan 5 software (Review Manager, The Cochrane Collaboration 2011). Relative risk with 95% CI was computed using a fixed effects model when heterogeneity was low and a random effects model otherwise. Funnel plots were constructed to assess publication bias.

Descriptive statistics were computed using RStudio Team (2022). RStudio: Integrated Development Environment for R. RStudio, PBC, Boston, MA, with *p* values < 0.05 considered significant. Between-group comparisons were performed by applying the chi-square test for qualitative data and Student’s test for quantitative data.

## 3. Results

The initial search identified 2405 eligible studies. After the elimination of duplicates, the predefined inclusion/exclusion criteria were applied, selecting 22 studies, including 1101 patients. A total of 506 had BT and 595 had PCR-BT (Figure 1: Flow chart). There were two comparative studies comparing BT with PCR-BT [15,22]. Only one randomized control trial was included [15]. The mean follow-up was 4.6 ± 1.1 years (range, 2–12 years). Outcome evaluations are shown in Supplementary Tables S1 and S2. We distinguished 2 different cohorts in 1 study and named Di Benedetto* group A with a follow-up of 6.5 years and Di Benedetto group B with a follow-up of 3 years [23]. No significant differences were found between sex, age, follow-up time, and fatty infiltration. In the BT group, there were statistical differences with more subscapularis lesions, more massive and ≥3 tendon tears, and arthritis ≥ grade 1 (Hamada and Fukuda) (Table 1). We had only a moderate- to high-quality studies MINORS score, and the mean score was 13.2 ± 3.2 (range, 9–14) (Supplementary Table S3).

### 3.1. Functional Evaluation

#### PCR-BT

**Figure 2:**
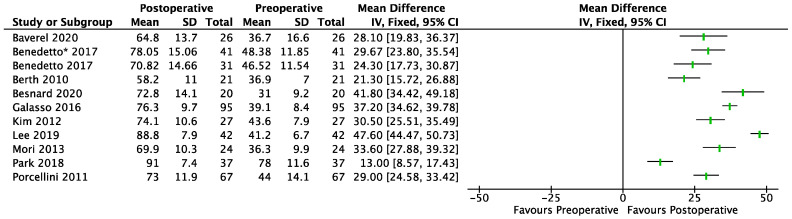
Forest plot of comparison: CMS (Constant-Murley Score), outcome in PCR-BT. SD: Standard Deviation. Di Benedetto * follow-up of 6.5 years; Di Benedetto follow-up of 3 years.

A total of 10 primary studies (cases series = 6, case–control = 3, randomized control trial = 1) [15,22,23,24,25,26,27,28,29,30,31] involving 431 shoulders evaluated improvements in the CMS at the final follow-up after PCR-BT (range, 13, 47.6) (Figure 2). The minimum preoperative and maximum postoperative CMS was respectively 31 and 88.8. Regarding the ASES, for 6 studies (cases series = 3, case–control = 3) [24,29,30,32,33,34] involving 183 shoulders, significant improvements in the ASES were demonstrated at the final follow-up (range, 31.8, 44.8) (Figure 2).

### 3.2. BT

**Figure 3:**
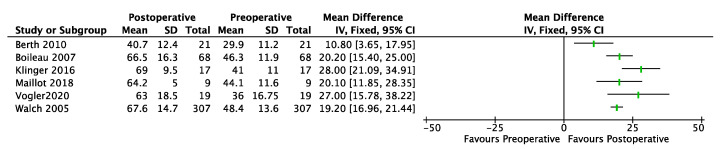
Forest plot of comparison: CMS (Constant-Murley Score), outcome in BT. SD: Standard Deviation.

A total of 6 primary studies for BT (cases series = 2, case–control = 2, randomized control trial = 1, prospective cohort study = 1) [15,22,35,36,37,38] involving 441 shoulders demonstrated significant improvements in the CMS at the final follow-up (range, 10.8,28) (Figure 3). The minimum preoperative and maximum postoperative CMS were, respectively, 29.9 and 67.6. For BT, 2 studies (cases series = 1, case–control = 1) [37,39] involving 50 shoulders demonstrated significant improvements in the ASES at the final follow-up (range, 30, 45.8).

### 3.3. Pain (VAS)

**Figure 4:**
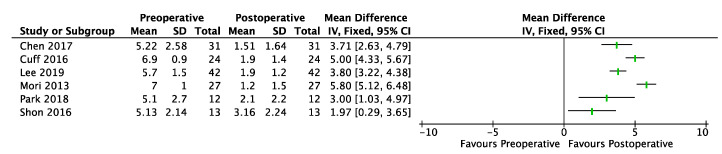
Forest plot of comparison: VAS, outcome in PCR-BT. SD: Standard Deviation.

**Figure 5:**
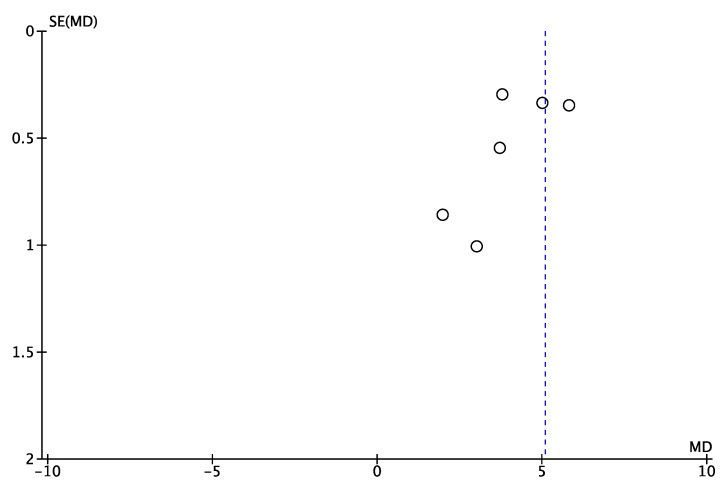
Funnel plot of comparison: VAS, outcome in PCR-BT. SE: Standard Error; MD: Mean Difference.

A total of 6 studies for PCR-BT (cases series = 4, case–control = 2) [22,28,29,30,32,33,34] involving 186 shoulders reported significant improvements at the final follow-up (range, 2, 5.8) (Figure 4 and Figure 5). 

**Figure 6:**
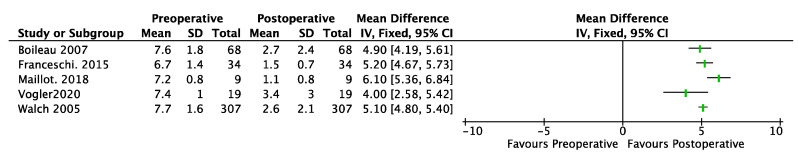
Forest plot of comparison: VAS, outcome in BT. SD: Standard Deviation.

**Figure 7:**
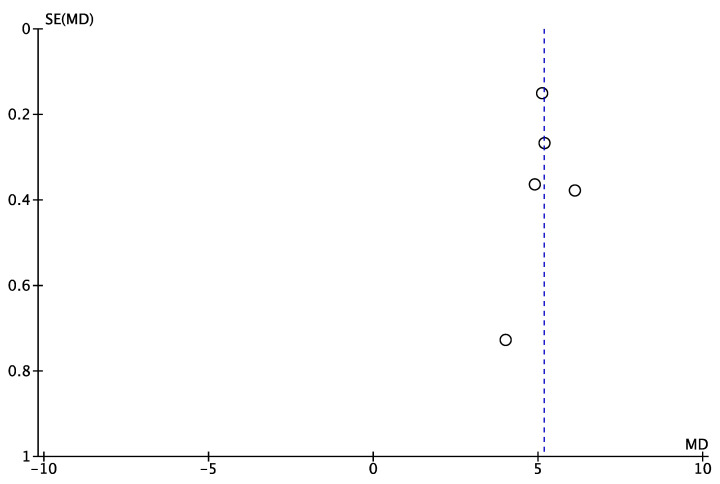
Funnel plot of comparison: VAS, outcome in BT. SE: Standard Error; MD: Mean Difference.

For BT, the results from 5 studies (cases series = 2, case–control = 2, prospective cohort study = 1) [16,22,35,37,38] involving 437 shoulders demonstrated significant improvements at the final follow-up (range, 4, 6.1) (Figure 6 and Figure 7).

### 3.4. Shoulder Motion (Forward Flexion, External Rotation, Abduction)

Regarding PCR, the results demonstrated wide improvements in forward flexion [15,16,22,23,24,25,28,29,30,33,40] (range, −14°, 59.4°) and abduction [15,23,28] (range, 35.3°, 60.2°), and low improvements in external rotation [15,16,22,24,28,29,30,33,40] (range, 1°, 19.1°). 

In the BT group, the 5 studies demonstrated improvements in forward flexion [15,16,22,35,38] (range, 2°, 27.9°) and negative influence over external rotation [15,16,22,35,38] (range, −8.6°,7°). Only 1 study (randomized control trial = 1) on abduction [15] reported an increase of 10° (range, −8.8°, 28.8°).

### 3.5. Acromiohumeral Distance (AHD)

For PCR-BT, the results of 5 studies (cases series = 5) [27,28,31,32,34] including 198 shoulders showed diminution of AHD at the last follow-up (range, −0.8–3 mm). Results from 4 BT studies (cases series = 3, case–control = 1) [35,37,38,39] with 416 shoulders showed diminution of AHD at the last follow-up (range, −4, −1.1). 

### 3.6. Complications

Thirteen studies reported complication and revision rates. In the BT group, 7 of the 8 studies (cases series = 3, case–control = 2, randomized control trial = 1, prospective cohort study = 1) [15,22,35,36,37,38,39] reported only 1 complication, a postoperative acute infection which was successfully cleared with antibiotics and arthroscopic irrigation [35]. Reported conversion rates were one hemi-arthroplasty at 20 months for severe glenohumeral arthritis [15], 1 RSA at 36 months for a missed preoperative osteoarthritis diagnosis [35], and 5 RSAs reported by 2 authors after a mean time of 63 months [37] (range, 45–97 months) for PCR-BT and 50 months [38] (range, 36–83 months) for BT. 

The authors reported complications in 7 of 16 studies [15,24,26,29,30,31,33]. A total of 6 of these complications were unspecified [31], and 1 involved repeat arthroscopic bursectomy and revision acromioplasty at 17 months due to persistent postoperative pain. A total of 2 studies [26,33] reported RSA conversion rates due to unsatisfactory outcomes, and 3 occurred at postoperative time points of 13 months, 30 months, and 56 months [33]. In the study by Galasso et al. [26], 6 had an RSA and 2 had a latissimus dorsi tendon transfer after a mean follow-up of 61.6 months (SD = 34.3 range, 24–121). Out of the 231 cases available, the overall conversion rate to arthroplasty or tendon transfer was 7.8%, with a rate of 8.9% (11/123) for PCR-BT and 6.4% (7/108) for BT.

## 4. Discussion

For irreparable MRCT, this systematic review demonstrated good outcomes in a large cohort of patients, regardless of whether isolated biceps tenotomy/tenodesis (BT) or arthroscopic partial repair with associated biceps tenotomy/tenodesis (PCR-BT) was performed. All studies documented significant improvements in subjective scores (Constant-Murley and ASES) and pain at the last follow-up. However, there was a trend toward greater improvement in the PCR-BT group. This may have been influenced by the severity of the cases in the BT group in terms of subscapularis tear, size, number of tendons involved, and osteoarthritis.

PCR-BT is published more often nowadays, and perhaps performed because people have more experience and desire to repair at any cost. To our knowledge, there are only two comparative studies on PCR-BT and BT for irreparable MRCT. In one such study, Berth et al. reported a good or satisfactory outcome according to PCR-BT. Regardless of high rates of structural failure of PCR-BT, the results of PCR-BT showed a slightly better functional outcome than BT. Franceshi et al. also showed that both techniques were effective in reducing patients’ symptoms, with better functional outcomes for PCR-BT.

In this systematic review, both techniques demonstrated significant improvements in postoperative functional outcomes, although there was a trend towards a greater magnitude of improvement in PCR-BT over BT in CMS (ranges: 13, 47.6 versus 10.8, 28). Both techniques exceed the MCID of 10 points [19,20], thus representing a noticeable change for the patient. In terms of ASES, there is a similar improvement for the BT group as for the PCR-BT group (ranges: 30, 45.8 and 31.81, 44.8, respectively. The improvements are greater than the MCID, but no difference between the two techniques seems to appear. As for pain, the improvements for both techniques are −5 points, much higher than the MCID. PCR-BT and BT reach the functional MCID threshold. Regarding the range of motion, a similar increase of +20° in forward flexion was observed, with negative evolution over external rotation for BT and improvement with (range, 1°, 19.1°) for PCR, and (range, 35.3°, 60.25°) for PCR-BT in abduction.

In the current study, we were not able to demonstrate, contrary to head-to-head comparisons [15,16,22], that functional score and pain using PCR-BT were improved compared to BT. 

Moreover, it is important to observe that there were differences between the two populations—in subscapularis tears, for instance. High rates of retear have been reported after arthroscopic repair when a subscapularis tear is present, but it does not seem to affect the functional scores [41,42]. Therefore, there were more instances with three tendons involved in the BT, and studies show that there were no significant differences in postoperative outcomes according to the different RCT patterns [26,43]. The fatty infiltration was similar in the two populations, and this parameter appears to be correlated with poor functional outcomes [44,45,46,47]. We had a large difference in terms of osteoarthritis in our populations, with more than 50% of Hamada ≥ 2 for BT and only 10% for PCR-BT. Glenohumeral arthrosis correlates with poor functional outcomes after surgery [48] and worsens the outcome in the BT population. 

Our main limitation is the lack of high-quality evidence studies on the treatment of irreparable MRCT. (1) There were only two comparative studies, both of which compared isolated biceps tenodesis or tenotomy to partial repair; the majority were cases series, and a few were case–control. We included nonrandomized studies because no randomized studies have been published. This is also an opportunity to examine the rationale for conducting a randomized trial by explicitly assessing the weaknesses of previous studies. This increases the risk of selection bias and confounding. The small number of published studies increases the risk of publication bias in these procedures. (2) We could not fully analyze complications and conversion rates to RSA because of insufficient data in the studies. Only 12 out of 22 reported these data, which cannot allow us to conclude as to the absence of complications or conversion during the mean follow-up of 4.5 years. (3) We observed different definitions of massive and irreparable rotator cuff; most of the time, MRCT was defined as ≥5 cm or ≥ two tendons involved. On the contrary, irreparable was defined in perioperative findings as a tear which cannot be fully repaired to its insertion. This could be a possible source of heterogeneity between studies. (4) The two populations were not fully comparable, with more arthritis in the BT population. (5) We also observed inconsistent patient reporting outcomes (PROs): CMS (76%; 18/24 studies), ASES (34%; 8/25 studies), pain scores (42%; 11/24 studies), and range of motion (79%; 19/24 studies). Satisfaction was only available for five studies; the strength was available for four studies. Other PROs were in the minority: UCLA (6 studies), SST (6 studies), SSV (2 studies), and DASH (2 studies). Regarding healing cuff repair, only 3 out of 16 studies mentioned their results. About revision surgery, only 5 out of 24 studies mentioned it. (6) Regarding acromioplasty, it is not always reported; we chose not to differentiate it, but it could transform a painful functional shoulder into a nonfunctional shoulder by disturbing the vertical balance.

We believe that our study has many potential strengths. First, there is no systematic review or meta-analysis that examines PCR-BT and BT as the main procedures for patients with pain and irreparable MRCTs. In this regard, there is a gap in clinical knowledge. Second, the last follow-up was at least two years, with a mean of almost five years, which could provide mid-term results of treatment. Third, unlike other studies, we focus on patients with severe preoperative pain (VAS > 5/10) and with a preserved range of motion (forward elevation > 100° and external rotation > 30°). Patients above 65 years old are considered on the edge for the RSA. The major challenges are pain relief and not deteriorating horizontal muscle balance [11].

Choosing the right treatment for patients with irreparable MRCT is challenging. It is critical to choose the best surgical option for the patient, assess functional impairment, and differentiate between this and pain. Retears in rotator cuff repair are more frequent in partial repair than complete repair, around 50% and 20%, respectively [49,50]. Retears primarily occur between six and twenty-six weeks after repair [51]. The worst treatment is one that turns a painful functional shoulder into a nonfunctional shoulder. Authors demonstrated significant worst outcome when retears, a partial repair improves functional outcomes but may make the patient worse [29]. Fatty infiltration was found to be a risk factor for enlarged retear [52]; other factors were increased levels of total cholesterol and low-density lipoprotein [52,53]. Although we did not compare the postoperative protocol, we can assume that PCR-BT is more demanding, with longer immobilization and rehabilitation, and more immediate postoperative pain than BT. Isolated biceps tenotomy/tenodesis may also improve, but may not worsen, functional outcomes. 

## 5. Conclusions

This systematic review suggests that PCR-BT or BT for MIRCT improves functional outcomes and reduces pain at midterm follow-up. Improvements in function and range of motion were slightly better with PCR-BT, and pain was better relieved with the BT procedure, but their clinical relevance is questionable. At this time, it is not possible to make a recommendation for or against treatment. Randomized trials comparing conservative treatment with isolated biceps tenotomy/tenodesis are needed to improve the understanding of irreparable MRCTs.

## Figures and Tables

**Table 1:** Comparison between the groups BT and PCR.

	BT		PCR		
n =	506		590		
Male	237	46.8%	312	52.9%	NS
Female	269	53.2%	278	47.1%	
Age (mean ± SD)	67.6	±5.9	6.5	±7.7	NS
Follow-up (mean ± SD)	4.7	±1.1	4.5	±1.2	NS
Subscapularis					<0.001
Normal	265	65.9%	480	85.3%	
Partial tear	76	18.9%	81	14.4%	
Complete tear	61	15.2%	2	0.4%	
Cuff tear Large	19	9.5%	51	13.5%	0.011
Massive	180	90.5%	328	86.5%	
Number of Tendons involved					<0.001
1	124	25.6%	19	4.8%	
2	219	45.2%	298	75.3%	
3	141	29.1%	79	19.9%	
Fatty infiltration (Goutallier)					0.324
1	2	1.5%	1	0.4%	
2	18	13.6%	30	12.3%	
3	89	67.4%	150	61.7%	
4	23	17.4%	62	25.5%	
Osteoarthritis (Grade)					<0.001
0	9	1.9%	225	56.3%	
1	233	49.4%	136	34.0%	
2	181	38.3%	30	7.5%	
≥3	49	10.4%	9	2.3%	

n: number; NS: not significant; SD: Standard Deviation; BT: Biceps Tenotomy or Tenodesis; PCR: Partial Cuff Repair.

## Supplementary Materials

The following supporting information can be downloaded at: https://www.mdpi.com/article/10.3390/jcm12072565/s1. Table S1. Clinical and radiological outcomes of BT. Table S2. Clinical and radiological outcomes of PCR. Table S3. Quality analysis of studies on MINORS criteria.
