# Supplementary material for: Outcomes of Isolated Biceps Tenodesis/Tenotomy or Partial Rotator Cuff Repair Associated with Biceps Tenodesis/Tenotomy for Massive Irreparable Tears: A Systematic Review
Source: J Clin Med. 2023 Mar 29;12(7):2565. doi: 10.3390/jcm12072565 (PMC10095538; doi:10.3390/jcm12072565)
Supplement: Supplementary file 1 [file jcm-12-02565-s001.zip › jcm-2264123 supplementary.pdf]

Table S1. Clinical and radiological outcomes of BT

BT: Biceps Tenotomy or Tenodesis ; LOE: level of evidence ; SD: Standard Deviation ; PRE: Preoperative ; LF: last follow-up

CMS: Constant-murley score ; ASES: American Shoulder and Elbow Surgeons ; VAS: Visual Analogue Scale; AHD: Acromio-humeral distance

Type of intervention	Study	Study Type (LOE)	Age mean ± SD (years)	Follow-up mean ± SD (months)	CMS mean ± SD	ASES mean	VAS mean ± SD	Forward flexion mean ± SD (°)	AHD mean ± SD (°)
BT	Berth et al. 2010	Randomized controlled Trial (2)	64.3 ± 3.4	24.7 ± 19.9	PRE: 29.9 ± 11.2<br>LF: 40.7 ± 12.4<br>LF-PRE: 10.8		PRE: 8.5 ± 1.6<br>LF: 5.8 ± 2.6<br>PRE-LF: 2.7	PRE: 98.5 ± 39.5<br>LF: 126.2 ± 28.8<br>LF-PRE: 27.7	
BT	Boileau et al. 2007	Case series (3)	71 ± 6	35 ± 7	PRE: 46.3 ± 11.9<br>LF: 66.5 ± 16.3<br>LF-PRE: 20.2		PRE: 7.6 ± 1.8<br>LF: 2.7 ± 2.4<br>PRE-LF: 4.9	PRE: 132 ± 43<br>LF: 155 ± 33<br>LF-PRE: 23	PRE: 5.6 ± 2<br>LF: 4.5 ± 2<br>PRE-LF: 1.1
BT	Franceschi et al. 2015	Case-control (3)	62 ± 7.25	93.6 ± 27.6			PRE: 6.7 ± 1.4<br>LF: 1.5 ± 0.7<br>PRE-LF: 5.2	PRE: 104.1 ± 12.2<br>LF: 132 ± 23.2<br>LF-PRE: 27.9	
BT	Klinger et al. 2016	Case-control (3)	68	33	PRE: 41<br>LF: 69<br>LF-PRE: 28				
BT	Liem et al. 2008	Case series (4)	70.6 ± 6.6	47 ± 15.7		PRE: 24<br>LF: 69.8<br>LF-PRE: 45.8	PRE: 7.8<br>LF: 2.9<br>PRE-LF: 4.9		PRE: 8.3<br>LF: 7<br>PRE-LF: 1.3
BT	Maillot et al. 2018	Prospective Cohort Study (2)	60	37.9 ± 3.1	PRE: 44.1 ± 11.6<br>LF: 64.2 ± 5<br>LF-PRE: 20.1		PRE: 7.2 ± 0.8<br>LF: 1 ± 0.8<br>PRE-LF: 6.2	PRE: 120 ± 27.4<br>LF: 122 ± 29<br>LF-PRE: 2	
BT	Vogler et al. 2020	Case-control (3)	68 ± 6.25	145 ± 12	PRE: 36<br>LF: 63<br>LF-PRE: 27	PRE: 36<br>LF: 66<br>LF-PRE: 30	PRE: 7.4<br>LF: 3.4<br>PRE-LF: 4		PRE: 10<br>LF: 6<br>PRE-LF: 4
BT	Walch et al. 2005	Case series (4)	64.3 ± 10.5	57 ± 36	PRE: 48.4 ± 13.6<br>LF: 67.6 ± 14.7<br>LF-PRE: 19.2		PRE: 7.73 ± 1.6<br>LF: 2.66 ± 2.13<br>LF-PRE: 5.07	PRE: 153.4 ± 40.4<br>LF: 164.6 ± 25.4<br>LF-PRE: 11.2	PRE: 6.6 ± 2.7<br>LF: 5.3 ± 2.9<br>PRE-LF: 1.3

Table S2. Clinical and radiological outcomes of PCR
PCR: Partial Cuff Repair ; LOE: level of evidence ; PRE: Preoperative ; LF: last follow-up
CMS: Constant-murley score ; ASES: American Shoulder and Elbow Surgeons ; VAS: Visual Analogue Scale; AHD: Acromio-humeral distance

Type of intervention	Study	Study Type (LOE)	Age mean ± SD (years)	Follow-up mean ± SD (months)	Constant mean ± SD	ASES Mean	VAS mean ± SD	Forward flexion mean ± SD (°)	AHD mean ± SD (°)
PCR	Baverel et al. 2020	Case-control (3)	65.8 ± 9	23.4 ± 3.5	PRE: 36.7 ± 16.6<br>LF: 64.8 ± 13.7<br>LF-PRE: 28.1	PRE: 33.5 ± 16.4<br>LF: 78.3 ± 19.3<br>LF-PRE: 44.8		PRE: 137.7 ± 30.5<br>LF: 158.1 ± 19.4<br>LF-PRE: 20.4	
PCR	Di Benedetto* et al. 2017	Case series (4)	67.21 ± 5.84	79.44 ± 15.96	PRE: 46.52 ± 11.54<br>LF: 70.82 ± 14.66<br>LF-PRE: 24.3			PRE: 96.33 ± 32.21<br>LF: 140 ± 36.48<br>LF-PRE: 43.67	
PCR	Di Benedetto et al. 2017	Case series (4)	67.93 ± 8.29	33.6 ± 12.12	PRE: 48.38 ± 11.85<br>LF: 78.05 ± 15.06<br>LF-PRE: 29.67			PRE: 81.38 ± 33.98<br>LF: 151.25 ± 26.11<br>LF-PRE: 69.87	
PCR	Berth et al. 2010	Randomized controlled Trial (2)	62.5 ± 2.3	23.8 ± 1.9	PRE: 36.9 ± 7<br>LF: 58.2 ± 11<br>LF-PRE: 21.3		PRE: 8.3 ± 1.6<br>LF: 4 ± 3.4<br>PRE-LF: 4.3	PRE: 105.3 ± 39.1<br>LF: 145.2 ± 28.1<br>LF-PRE: 39.9	
PCR	Besnard et al. 2020	Case series (4)	59.3 ± 8.4	96.7 ± 7	PRE: 31 ± 9.2<br>LF: 72.8 ± 14.1<br>LF-PRE: 41.8		PRE: ±<br>LF: 1.1 ± 1.5<br>PRE-LF: -1.1	PRE: 132 ± 42.9<br>LF: 176 ± 16<br>LF-PRE: 44	
PCR	Burkhart et al. 2007	Case series (4)	66.5 ± 9.26	39.3 ±				PRE: 103.2 ± 46.3<br>LF: 156.9 ± 32.7<br>LF-PRE: 53.7	
PCR	Chen et al. 2017	Case series (4)	60.3 ± 6.3	29.6 ± 6.6		PRE: 45.95 ± 20.56<br>LF: 78.59 ± 14.29<br>LF-PRE: 32.64	PRE: 5.22 ± 2.58<br>LF: 1.51 ± 1.64<br>PRE-LF: 3.71		PRE: 8.32 ± 2.74<br>LF: 8.78 ± 2.53<br>PRE-LF: -0.45
PCR	Cuff et al. 2016	Case series (4)	65.2	71.1		PRE: 46.6 ± 6.9<br>LF: 79.3 ± 7.8<br>LF-PRE: 32.7	PRE: 6.9 ± 0.9<br>LF: 1.9 ± 1.4<br>PRE-LF: 5	PRE: 168 ± 70<br>LF: 154 ± 29<br>LF-PRE: -14	
PCR	Franceschi et al. 2015	Case-control (3)	62 ± 7.25	93.6 ± 27.6			PRE: 6.8 ± 1.6<br>LF: 0.9 ± 1.1<br>PRE-LF: 5.9	PRE: 111.5 ± 13.3<br>LF: 163.5 ± 12.9<br>LF-PRE: 52	

PCR	Galasso et al. 2016	Case series<br>(4)	62.7 ± 7.3	82.7 ± 37.1	PRE: 39.1 ± 8.4<br>LF: 76.3 ± 9.7<br>LF-PRE: 37.2			PRE: ±<br>LF: 172 ± 17.6<br>LF-PRE: 172	
PCR	Kim et al. 2012	Case series<br>(4)	62.3	41.3	PRE: 43.6 ± 7.9<br>LF: 74.1 ± 10.6<br>LF-PRE: 30.5				PRE: 6.5 ± 1.5<br>LF: 5.9 ± 1.8<br>PRE-LF: 0.6
PCR	Lee et al. 2019	Case series<br>(4)	61.2 ± 9.1	35.4 ± 7.3	PRE: 41.2 ± 6.7<br>LF: 88.8 ± 7.9<br>LF-PRE: 47.6		PRE: 5.7 ± 1.5<br>LF: 1.9 ± 1.2<br>PRE-LF: 3.8	PRE: 131.5 ± 49.6<br>LF: 166.8 ± 22.5<br>LF-PRE: 35.3	PRE: 6.5 ± 1.7<br>LF: 6.3 ± 1.6<br>PRE-LF: 0.2
PCR	Mori et al. 2013	Case-control<br>(3)	65.4 ± 9.2	35.7 ± 7	PRE: 36.3 ± 9.9<br>LF: 69.9 ± 10.3<br>LF-PRE: 33.6	PRE: 41.8 ± 11.3<br>LF: 85.7 ± 14.1<br>LF-PRE: 43.9	PRE: 7 ± 1<br>LF: 1.2 ± 1.5<br>PRE-LF: 5.8	PRE: 110.6 ± 34.2<br>LF: 162.3 ± 9.4<br>LF-PRE: 51.7	
PCR	Park et al. 2018	Case-control<br>(3)	63.8 ± 7.2	29.8 ± 8.7	PRE: 78 ± 11.6<br>LF: 91 ± 7.4<br>LF-PRE: 13	PRE: 51.5 ± 22.7<br>LF: 78.5 ± 18.5<br>LF-PRE: 27	PRE: 5.1 ± 2.7<br>LF: 2.1 ± 2.2<br>PRE-LF: 3	PRE: 143.2 ± 8.7<br>LF: 145.3 ± 5.9<br>LF-PRE: 2.1	
PCR	Porcellini et al. 2011	Case series<br>(4)	63	60	PRE: 44 ± 14.1<br>LF: 73 ± 11.9<br>LF-PRE: 29				PRE: 6.1 ± 1.6<br>LF: 9.1 ± 2.2<br>PRE-LF: -3
PCR	Shon et al. 2016	Case series<br>(4)	65.9 ± 6.5	40.5 ± 14.9		PRE: 41.97 ± 15.08<br>LF: 73.78 ± 21.55<br>LF-PRE: 31.81	PRE: 5.13 ± 2.14<br>LF: 3.16 ± 2.24<br>PRE-LF: 1.97		PRE: 6.91 ± 1.89<br>LF: 6.13 ± 2.12<br>PRE-LF: 0.78

Table S3. Quality analysis of studies on MINORS criteria.
n/a: not applicable ; 0: not reported ; 1: reported but inadequate ; 2: reported and adequate.

Authors	A stated aim of the study	Inclusion of consectuve patients	Prospective collection of data	Endpoint appropriate to the aim of the study	Unbiased evaluation of endpoint	Follow-up period appropriate to the major endpoint	Loss to follow-up less than 5%	Prospective calculation of the study size	An adequate control group	Contemporary group	Baseline equivalence of groups	Adequate statistical analysis	Total	Quality assessment
Baverel	2	2	1	2	0	2	0	2	n/a	2	2	2	17	Moderate
Benedetto	2	2	1	2	0	2	0	0	1	2	2	1	15	Moderate
Berth	2	1	2	2	0	2	0	0	2	2	2	2	17	Moderate
Besnard	2	2	1	2	0	2	0	2	n/a	n/a	n/a	2	13	High
Boileau	2	2	1	2	0	2	1	0	n/a	n/a	n/a	2	12	Moderate
Burkhart	2	1	1	2	0	2	0	0	n/a	n/a	n/a	2	10	Moderate
Chen	2	1	1	2	0	2	1	0	n/a	n/a	n/a	2	11	Moderate
Cuff	2	1	1	2	0	2	0	0	n/a	n/a	n/a	2	10	Moderate
Franceschi	2	0	2	2	0	2	2	2	2	2	2	2	20	High
Galasso	2	1	2	2	2	2	1	0	n/a	n/a	n/a	2	14	High
Kim	2	0	1	2	0	2	0	0	n/a	n/a	n/a	2	9	Moderate
Klinger	2	1	0	2	0	2	0	0	n/a	n/a	n/a	2	9	Moderate
Lee	2	0	1	2	2	2	1	0	n/a	n/a	n/a	2	12	Moderate
Liem	2	2	1	2	0	2	0	0	n/a	n/a	n/a	2	11	Moderate
Maillot	2	2	2	2	0	2	2	0	2	2	2	2	20	High
Mori	2	2	2	2	2	2	1	0	n/a	n/a	n/a	2	15	Moderate
Park	2	0	1	2	2	2	1	2	n/a	n/a	n/a	2	14	Moderate
Porcellini	2	2	1	2	0	2	1	2	n/a	n/a	n/a	2	14	Moderate
Shon	2	2	1	2	0	2	0	0	n/a	n/a	n/a	2	11	Moderate
Vogler	2	2	1	2	0	2	1	0	n/a	n/a	n/a	2	12	Moderate
Walch	2	2	1	2	0	1	1	0	n/a	n/a	n/a	2	11	Moderate
